# Excess of mutational jackpot events in expanding populations revealed by spatial Luria–Delbrück experiments

**DOI:** 10.1038/ncomms12760

**Published:** 2016-10-03

**Authors:** Diana Fusco, Matti Gralka, Jona Kayser, Alex Anderson, Oskar Hallatschek

**Affiliations:** 1 Department of Physics, University of California, Berkeley, California 94720, USA; 2 Department of Integrative Biology, University of California, Berkeley, California 94720, USA

## Abstract

The genetic diversity of growing cellular populations, such as biofilms, solid tumours or developing embryos, is thought to be dominated by rare, exceptionally large mutant clones. Yet, the emergence of these mutational jackpot events is only understood in well-mixed populations, where they stem from mutations that arise during the first few cell divisions. To study jackpot events in spatially structured populations, we track mutant clones in microbial populations using fluorescence microscopy and population sequencing. High-frequency mutations are found to be massively enriched in microbial colonies compared with well-shaken liquid cultures, as a result of late-occurring mutations surfing at the edge of range expansions. Thus, jackpot events can be generated not only when mutations arise early but also when they occur at favourable locations, which exacerbates their role in adaptation and disease. In particular, because spatial competition with the wild type keeps most mutant clones in a quiescent state, strong selection pressures that kill the wild type promote drug resistance.

## Abstract (web summary)

Large mutant clones arising from early mutations in growing cell populations facilitate short-term evolution in microbes and in tumours. Here the authors analyse spatially expanding colonies, and show that large mutant clones can also arise late when they surf at expanding frontiers.

## Graphical abstract

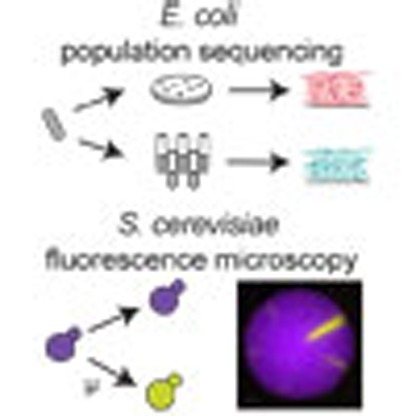

One of the hallmarks of spontaneous mutations in growing populations is the emergence of mutational jackpot events—large mutant clones arising from mutations that by chance occur early in the development of a cellular population so that their progenitors benefit from prolonged growth. Due to their sheer size, these jackpot events, first discovered by Luria and Delbrück^1^, are thought to have momentous roles in short-term evolutionary processes, including the adaptation from standing variation^2^,^3^,^4^, evolutionary rescue^5^, drug resistance evolution^6^,^7^,^8^,^9^,^10^ and the somatic evolution of genetic diseases^11^,^12^. However, because the emergence of jackpot events has been understood only in uniformly growing populations^1^,^10^,^13^, it is currently impossible to predict their impact on the evolution of many naturally structured populations.

The original Luria–Delbrück experiment studied mutant clones arising in well-mixed microbial populations and detected the size of resistant clones by counting single colonies on selective plates. Here, we generalize the assay in two ways: (i) by studying mutant clones arising in spatially structured populations and (ii) by using a combination of next-generation sequencing and fluorescence microscopy techniques to accurately detect size and structure of high-frequency clones.

We find that high-frequency clones are massively enriched in microbial colonies compared with well-shaken liquid cultures, as a result of late-occurring mutations surfing at the edge of range expansions^14^,^15^,^16^. We provide a mathematical theory that explains the observed excess of jackpot events and predicts their role in promoting rare evolutionary outcomes. In particular, we show that resistant clones generated by surfing can become unleashed under high selection pressures, and thus represent a drug resistance hazard for high-dose drug treatments. In this context, our theory offers an innovative explanation for the phenomenon of ‘competitive release', initially observed in ecology^17^,^18^,^19^, and more recently in tumour evolution^20^, where the craved resource is space rather than nutrients.

## Results

### Generalized Luria–Delbrück experiments

**Figure 1:**
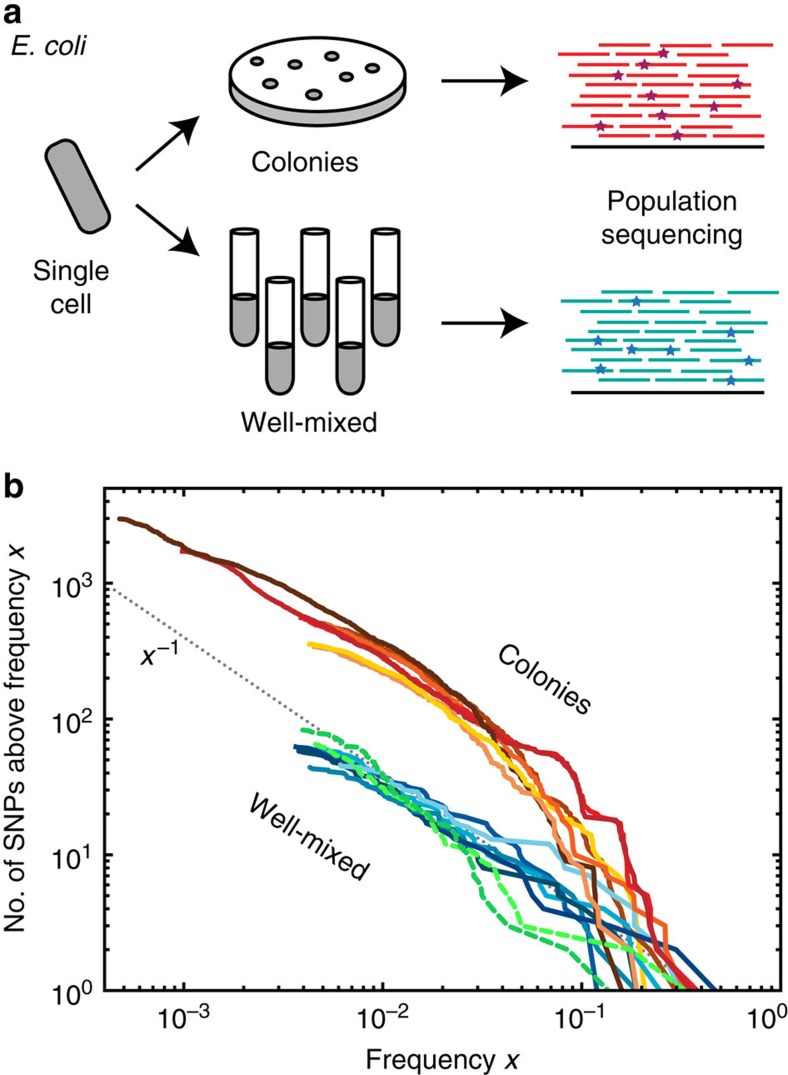
Population sequencing reveals an excess of jackpot events in spatially growing populations. (**a**) Starting from few cells of a mutator strain of the bacterium *E. coli* (*mutT* deletion, see Methods section), we grew 6 colonies and 6 liquid cultures up to an average population size of 3 × 10^9^ cells and sequenced each population at a coverage of at least 1000 × (Supplementary Table 1). The number of SNPs that occurred at a frequency higher than *x* is displayed in panel (**b**) (solid lines: colonies in warm colours, well-mixed in cold colours; dashed lines: well-mixed on plates). We found about 10 times more mutants above a frequency of 1% in colonies than in well-mixed population, even when the latter were grown on plates (Methods). The dotted black line is the fit to the well-mixed data with a mutation rate of *μ*=0.4 per genome per replication.

To measure the size of mutational jackpot events, we employed population sequencing with low error rates (see Methods section), which returned frequencies of new mutations at many genomic sites simultaneously and independently of their phenotypic effect. Specifically, we sequenced populations of a mutator strain of *Escherichia coli* cultured in well-mixed liquid medium, where growth is uniform, and as colonies on solid agar medium, where most growth occurs at the colony edge (see Methods section)^21^,^22^. By growing from a small number of initial cells to a similar final size, all populations went through a comparable number of cell divisions (between 1 × 10^9^ and 7 × 10^9^, Supplementary Table 1). Counting the observed frequencies of Single Nucleotide Polymorphisms (SNPs) in the populations, we obtained the number of sites in the genomes where the clonal sub-population carrying the derived mutation had a frequency larger than a given frequency value *x*, shown in Fig. 1b. Our deep sequencing procedure allowed us to detect all clones that have frequencies larger than about 10^−3^ (see Methods section), yielding around 600 such high-frequency events in each colony, which characterize the statistics of jackpot events.

Populations of the same size and mutation rate are expected to experience the same total number of mutational events, on average, independently of the mode of growth (liquid versus solid medium). Yet, Fig. 1 shows that colonies had approximately ten times more mutant clones above frequencies of 1%—corresponding to clones of at least 10^7^ cells—than well-mixed populations. This difference cannot be explained by the variation in final population size, since some well-mixed populations had a larger final size than some colonies (Supplementary Table 1, Supplementary Fig. 1).

To test whether the discrepancy was caused by different mutation rates in liquid culture and on agar plates, we also sequenced ‘plated well-mixed' populations whose growth was kept uniform by regularly spreading the cells across the plate (see Methods section). The resulting distribution of SNPs is consistent with the well-mixed populations in liquid culture (green dashed and blue solid lines in Fig. 1b), confirming that the mutation rate is not significantly affected by the mode of growth (see also Supplementary Methods and Supplementary Table 2). Thus, we conclude that the observed difference in the clone size distribution must be a consequence of the non-uniform growth in colonies, which results in a surprising number of mutations at high frequency.

### Fluorescence microscopy reveals spatial structure of clones

**Figure 2:**
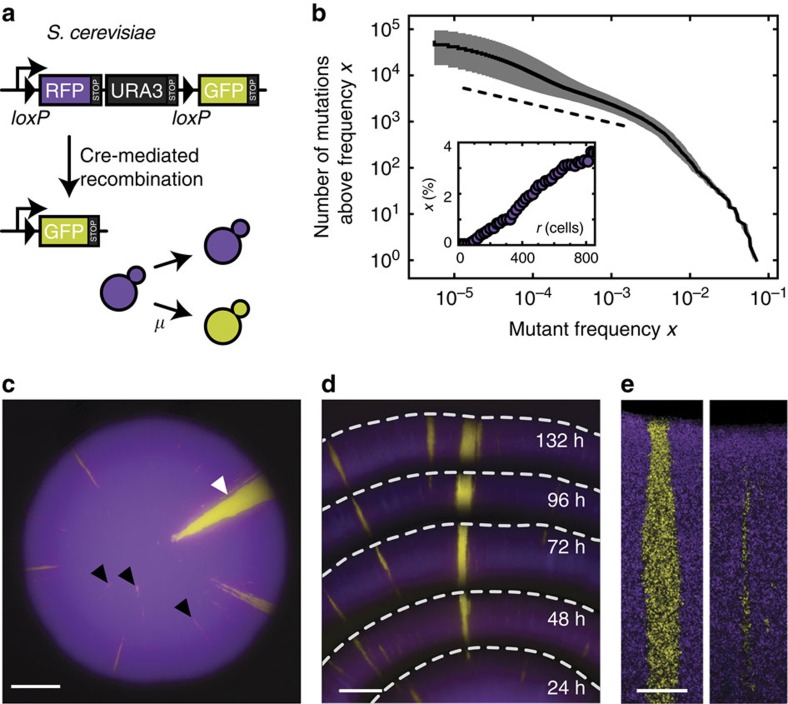
Visualizing mutational jackpot events in colonies. (**a**) An engineered strain of *S. cerevisiae* that stochastically switches from RFP (purple) to GFP (yellow) at a rate of 1.6 × 10^−3^ per cell division enables us to visualize high frequency jackpot events as they arise during colony growth (**c**, scale bar, 1 mm). (**b**) Image analysis (see Methods section) of mutant clones in 343 colonies reveals a shoulder-like distribution of clone sizes, roughly consistent with our predictions for idealized 2D populations (dashed line, see also Methods section). The inset shows the median mutant frequency as a function of radius *r*. Error bars are smaller than symbols. (**d**, scale bar, 0.5 mm) Monitoring the spatial distribution of mutants using fluorescence time-lapse microscopy (see also Supplementary Movie 1) reveals that mutant clones come either as sectors^21^ with actively growing front regions (**e**, left, scale bar, 0.2 mm; **c**, white arrow) or as ‘bubbles' (**e**, right; **c**, black arrows), which are non-growing mutant clones that have lost contact with the expanding edge.

To study the nature of the high-frequency clones in colonies, we monitored the spatial distribution of mutant clones using separate fluorescent marker experiments. We employed a genetically engineered budding yeast strain capable of switching from a red-fluorescing state to a green-fluorescing state at a rate of about 1.6 × 10^−3^ per replication^23^. This heritable, non-reversible switch is mediated by the stochastic expression of Cre recombinase (see Fig. 2a and Methods section for details). As shown in Fig. 2c, the resulting colonies exhibited both elongated speckles (dark arrows), which we termed ‘bubbles', as well as previously described spoke-like sectors (white arrow)^21^. Importantly, image analysis of the clone area obtained from 343 colonies yielded a histogram consistent with the shoulder-like distribution obtained from our sequencing approach (Fig. 2b and Methods section). Thus, both the fluorescent data from just one ‘engineered' site and the sequencing data covering many genomic sites seem to reflect the same mechanism shaping the clone size distribution.

The fluorescence data, moreover, revealed where clones emerge and how they grow. Time-lapse movies showed that most high-frequency clones first arise near the front of the growing colony (Fig. 2e, Supplementary Movie 1). The resulting clonal patches grow with the advancing frontier until they lose contact to the front, whereupon they become trapped as bubbles in the non-growing bulk of the population. Rarely, clones are able to ‘surf' at the front until the end of the experiment and give rise to sectors. Such allele surfing is a characteristic feature of range expansions^14^,^16^,^24^ and has been demonstrated to be pervasive in microbial communities^21^,^25^,^26^,^27^.

### Gene surfing theory explains mutational jackpot events

**Figure 3:**
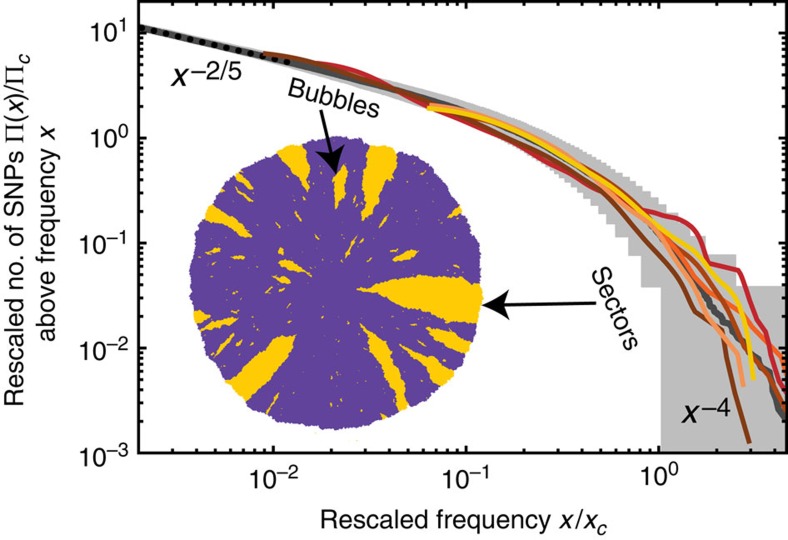
Simulations of range expansions reproduce the measured distribution of clone sizes. The clone size distribution obtained from our meta-population simulations (grey solid line) exhibits two predictable power-law tails that characterize bubbles (dotted line) and sectors, respectively (see Methods section). The experimental mutant spectra (coloured lines) collapse onto this theory line upon scaling the two axes by the frequency of the largest bubble *x*_*c*_ and the probability Π_*c*_ of observing a clone larger than *x*_*c*_, respectively (the shaded area represents the 95% confidence interval, see Methods section for details).

To understand how gene surfing generates clones of different sizes, we studied their emergence in range expansion simulations. Specifically, colony growth was implemented by the random addition of new demes to the advancing frontier. The newly added deems inherited their ancestral genotype unless they mutated, which occurred at a fixed rate (see Methods section; Supplementary Movie 2). Interestingly, this simple meta-population model generated a clone size distribution that accurately reproduced the measured one, as can be seen in Fig. 3. Our simulation results, covering over four orders of magnitude, also reveal that the distribution crosses over between two power-law distributions with distinct exponents. Analysis of the clone shapes produced in our simulations shows that the power-law regimes of low and high frequencies characterize bubbles and sectors, respectively.

**Figure 4:**
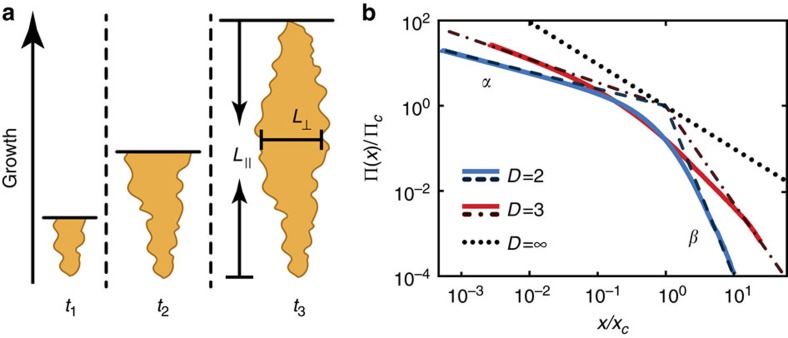
Fractal properties of clone boundaries control the size distribution of bubbles. (**a**) Sketch of the stochastic growth of a bubble, which is bounded by two annihilating random walks. (**b**) Rescaled clone size distribution from simulations in two and three dimensions approach the predicted asymptotic power-law behaviour for bubbles, *x*^−*α*^, and sectors, *x*^−*β*^, in the limit of small and large frequencies, respectively (dashed lines: asymptotic theory predictions). The values of the exponents are reported in Table 1.

The effect of gene surfing is not limited to two-dimensional (2D) growth: simulations of spherically growing meta-populations (Fig. 4b), such as those used to model solid tumours^28^,^29^, still result in mutant spectra with two distinct power-law regimes corresponding to bubbles and sectors.

The power-law exponents can be derived analytically by treating the boundaries of the mutant clones as annihilating random walks. The statistical properties of these random walks determine the relationship between the length *L*_||_ of clones parallel to the growth direction and the corresponding perpendicular length *L*_⊥_ (see Fig. 4a). For instance, purely diffusive clone boundaries, which occur when the population front is completely flat, result in 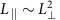; more generally, 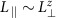, where the dynamical exponent *z* depends on the details of how the population grows (dimensionality and roughness of the growing front). The Kardar–Parisi–Zhang (KPZ) interface growth model^30^, which has been found consistent with bacterial growth patterns^21^, predicts *z*_2*D*_=3/2 (exact) and *z*_3*D*_=1.63 (numerical)^31^. By computing the area enclosed between two annihilating random walks, which are unbiased in the neutral case, one can determine the power-law exponent for bubbles (see Methods section). The power-law exponent for sectors follows from the one for bubbles via an exact scaling relationship (see Methods section). The results for different scenarios are summarized in Table 1.

Importantly, our scaling arguments predict that the clone size distributions obtained for different population sizes and mutation rates should collapse onto one master curve when the clones frequencies are measured in terms of the characteristic frequency of the largest occurring bubble *x*_*c*_, and the clone number in terms of the average probability for a new mutation to establish a sector, Π_*c*_. Indeed, after rescaling, our sequencing data show remarkable agreement with the master curve obtained from our simulations (Fig. 3, Supplementary Methods, Supplementary Table 3, and Supplementary Fig. 2).

### Role of jackpot events in the evolution of drug resistance

One of the striking features of the clone size distribution under range expansion is the excess of high frequency mutations over the well-mixed expectation. The primary consequence of more jackpot events is that, typically, the total number of mutants will be much larger in a spatially growing population compared with an equally large well-mixed population. This can be understood from the following simple mathematical argument.

**Figure 5:**
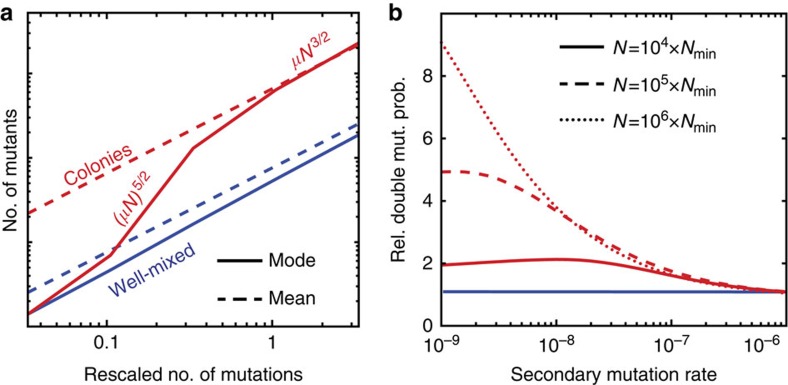
Gene surfing theory predicts an excess of jackpot events and its consequences. (**a**) Sampling the experimental clone size distribution Π, we found that the typical (mode, solid line) and the mean (dashed line) number of mutants as a function of the rescaled number of mutations *μN*Π_*c*_, which corresponds to the number of mutations establishing into sectors, is higher in range expansions (red) than in equally large uniformly grown populations (blue). (**b**) The excess of large-frequency clones promotes multi-step evolutionary processes, such as the emergence of double mutants. The relative probability (compared with the well-mixed expectation) of producing a double mutant is always higher in a colony, especially when the secondary mutation rate is low. Here, *N*_min_=*x*_min_*N* (see Methods section), which in our *E. coli* colonies corresponds to *N*_min_≈10^5^.

If mutations arise at a low rate *μ* per cell division as the population is growing to a final size *N*, one expects *μN* mutational events to occur. In each generation, the frequency of mutants in the population increases by *μ*, on average. In a well-mixed uniformly grown population, the number of generation is log_2_*N*, and hence the expected total number of mutants in this case is proportional to *μN* log_2_*N*. In a range expansion, only cells near the edge of the colony have access to sufficient nutrients, leading to the formation of a layer of growing cells of width *λ* (in units of cell diameters). Since a length *λ* is added per generation to the radius of the colony, we can estimate that *R*/*λ*∝*N*^1/2^/*λ* generations elapse at the frontier during the growth process. Hence, the final total number of mutants created during a range expansion is proportional to *μN*^3/2^/*λ*, which for large *N* is much larger than in a uniformly grown population of the same size. Note that, as in the classic Luria–Delbrück case, the mean is usually not a useful quantity because it is dominated by very rare, large events. Nevertheless, both the typical and the mean number of mutations exceed the well-mixed expectations as our stochastic analysis shows (Fig. 5, Supplementary Note 1 and Supplementary Fig. 3).

Jackpot events can be key in acquiring complex phenotypes, such as drug resistance or the onset of cancer, which often require the accumulation of multiple mutations^11^,^32^. Range expansions may promote the acquisition of secondary mutations because the pool of individuals carrying the first (driver) mutation is, both on average and typically, larger than in uniformly grown populations (Fig. 5a). As shown in Fig. 5b, the probability of secondary mutations can be almost an order of magnitude larger in spatial populations compared with well-mixed ones, especially when mutation rates are low (Supplementary Note 2).

### Drugs can trigger competitive release of mutant clones

**Figure 6:**
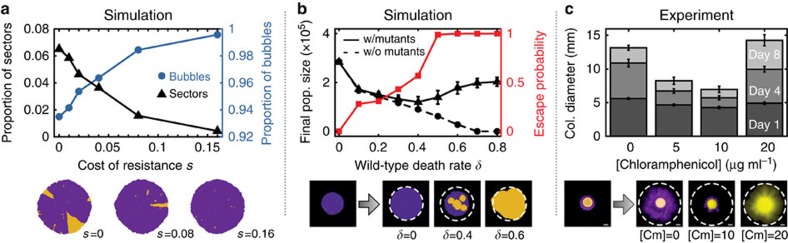
High drug concentration treatments can promote the spreading of resistance by unleashing mutants that are otherwise trapped within the colony bulk by competitive release. (**a**) When the mutants carry a cost *s* in the absence of antibiotics, the fraction of mutants trapped in bubbles is increased and sectors become increasingly rare. (**b**) Starting with (i) colonies pre-grown with *s*=0.4 (black triangles, solid line) or (ii) resistance-free control colonies (black circles, dashed line) as initial condition, we simulated the effects of treatment with antibiotics at varying concentrations by tuning the death rate *δ* of the wild type (see Methods section). After 140 generations, resistance-free colonies showed decreasing population size up to complete eradication for *δ*>0.7, as expected. In contrast, colonies with resistant bubbles exhibited an initial decrease in population size followed by a steady increase for *δ*>0.4 (error bars represent standard deviations over 100 simulations). Visualization of the simulated colonies showed that in this range of death rates, the previously encapsulated mutants escape the surrounding wild type (red squares) and can then grow indefinitely (Supplementary Movie 3). A high dose of antibiotics may thus not only fail to eradicate the population, but even promote the spreading of resistance. This effect can be reproduced in conceptual experiments with *E. coli*, shown in (**c**), which demonstrate that high antibiotic concentrations can release trapped mutant clones. A droplet of resistant cells (yellow) embedded in a larger droplet of susceptible cells (purple) was inoculated at different antibiotic concentrations (see Methods section). After 8 days of growth intermediate antibiotic concentrations exhibited the least amount of total population growth. The highest drug concentrations eradicated the wild type and thus allowed the resistant mutants to spread freely. Error bars represent standard deviations over 16 replicates and scale bars correspond to 2 mm.

Evolutionary dynamics is influenced by mutational jackpot events not only because of their size but also because of their particular spatial structure. The emergence of sectors, which sporadically arise from neutral mutations, is strongly suppressed when mutants carry a cost (Fig. 6a, Supplementary Note 3 and Supplementary Fig. 4), commonly observed for drug resistance in the absence of antibiotics^33^: deleterious mutants can surf only briefly before they are overtaken by faster-growing wild-type cells and fall behind the growing frontier.

In the absence of antibiotics, then, costly resistant clones are expected to reside in bubbles, encased by an expanding wild-type population. Upon a sudden environmental change, for example, by a strong antibiotic attack killing the susceptible wild type, the trapped mutants may become unleashed, regrowing and thus rescuing the population from extinction. This evolutionary rescue is brought about by a particular kind of competitive release, in which the indispensable resource is space. Competition in this case is extreme: trapped clones are not only at a disadvantage, but they have no chance of escaping unless the surrounding wild type vanishes. Consistently with this idea, we expect minimal net population growth and successful containment of resistant cells at intermediate drug concentrations, sufficiently strong to slow down proliferation of the wild type without eradicating it. Indeed, when we implemented drug treatment as a tunable death rate for wild-type cells (Fig. 6b, Supplementary Movie 3, see Methods section), our simulations showed the smallest net population growth for intermediate death rates. In contrast, high death rates not only failed to eradicate the population, but promoted the spread of resistance by generating an entirely resistant population.

Our simulations provide a uniquely spatial mechanism of how excessively high drug concentrations can promote the spreading of drug resistance. This effect can be reproduced in conceptual experiments in which we embedded resistant cells within a colony of susceptible cells. We found that the resistant cells stayed trapped even at intermediate drug concentrations that severely limit the wild-type growth. For higher drug concentrations, the mutants were released and grew rapidly (Fig. 6c). Hence, to optimally curtail microbial growth in our particular set-up, the drug concentration should indeed be set at an intermediate sweet spot.

## Discussion

In combination with a generalization of the Luria–Delbrück theory, our experiments show that the process of allele surfing generates an excess of high-frequency clones: mutations have a much higher chance of being carried by a high proportion of the population. These high-frequency clones come as growing sectors and non-growing bubbles, which are spatially encased by wild-type cells. Our theory suggests that the excess of jackpot events is not limited to microbial colonies but arises generally in populations that exhibit non-uniform growth rates in two or three dimensions.

In addition to antibiotic resistance evolution in pathogenic biofilms, an excess of jackpot events could thus be relevant also during the somatic evolution of some types of cancer^12^,^34^,^35^, as growing solid tumours often exhibit less growth in necrotic core regions^36^ and sectoring has been recently documented^29^. Moreover, it has been argued that jackpot events play a crucial role in the predisposition to cancer and other genetic diseases^11^. Based on the classic Luria–Delbrück theory, it was predicted that a large fraction of cancers may arise from predisposed stem-cell lineages^32^. Our results suggest that if growth is non-uniform during the development of the stem cell pool, predisposed stem-cell lines may occur even more frequently than previously hypothesized.

Our sequencing study design can be applied to test this prediction by measuring the site frequency spectrum in tumours as well as in multi-cellular organisms at different stages of their development. Some clues about large-frequency clones could also be collected from visible phenotypes resulting from somatic mosaicism, such as Blaschko's lines^11^. Plants or animals with pigmentations caused by mobile elements are particularly amenable to such pattern analyses.

Our experiments, simulations and theory not only found an excess of jackpot events but also elucidated their spatial patterns. Since drug resistance often comes at a selective cost in the absence of the drug, most resistance mutant clones are expected to be small and hidden in the bulk of the susceptible wild-type population. A high-dose treatment removes the wild type as the natural competitor of the resistant mutants and, consequently, triggers a competitive release of the dormant mutants. This form of competitive release is extreme in the sense that mutants stop growing entirely before the release, due to lack of space and nutrients. Because a broad spectrum of dormant clones is also generated in three-dimensional (3D) growth, this spatial form of competitive release can be particularly relevant in solid tumours. Indeed, the hypothesis that growth control may, under certain conditions, be more effective than attempts of complete eradication has been recently proposed in the context of cancer^37^,^38^ based on mathematical modelling of exponentially growing tumours treated with a varying dose of chemotherapeutic drugs over time. A recent study^20^ tested this hypothesis experimentally in a mouse model and found that prolonged chemotherapy with low doses was the most effective at keeping the tumour in check. In contrast, tumours that had regrown after an initial shrinkage following high-dose treatment did not respond to a second round of treatment, possibly indicating the emergence of resistance. Our results provide a novel mechanism, based on competition for space, that can explain these observations.

As sequencing costs continue to decrease, a growing number of studies utilizes population sequencing to draw conclusions on how cellular populations evolve. In this context, our results urge for caution when employing the classic Luria–Delbrück theory as a general theory for neutral evolution, as has been proposed in a recent meta-study of intra-tumour heterogeneity^12^. For one thing, spatial effects complicate the estimation of mutation rates and the amount of resistant tumour cells. More importantly, deviations from the classic Luria–Delbrück theory may simply indicate non-uniform growth rather than non-neutral evolution.

## Methods

### Scaling of clone size distribution in expanding populations

We consider a population expanding from one to *N* individuals without death. Our goal is to characterize the probability Π(*x*) that a mutation randomly introduced at the birth of one of the *N* cells generates a clone of frequency equal or larger than *x* (Fig. 1a). In other words, Π(*x*) is the reverse cumulative distribution of the size of clone introduced by a random mutation. The derivative −∂_*x*_Π(*x*) represents the population site frequency spectrum that can be obtained directly from population sequencing. Two extreme values of Π(*x*) are known *a priori*: Π(1/*N*)=1 because any mutation will certainly reach at least frequency 1/*N* if we ignore death. On the other hand, Π(1)=1/*N* because this requires the mutation to be introduced in the very first birth, that is, at the root of the genealogical tree.

The behaviour of Π(*x*) in between these boundary points is known in the well-mixed or infinite-dimensional case, studied by Luria and Delbrück; it is a single power law (*Nx*)^−1^ (ref. 13). Our experiments and simulations suggest that the finite-dimensional case is characterized by two asymptotic regimes: a low-frequency regime (*Nx*)^−*α*^ with exponent *α*<1, corresponding to bubbles, and a high-frequency regime *N*^−1^*x*^−*β*^ with exponent *β*>1, corresponding to sectors. The *N*-dependent pre-factors of both regimes are fixed by the boundary conditions, and the crossover point *x*_*c*_ follows from continuity (Supplementary Fig. 5). The described crossover behaviour can be captured mathematically by the scaling form


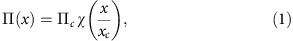


in terms of the crossover frequency 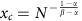 and a crossover probability scale 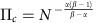. The scaling function *χ*(*ξ*) exhibits two power-law regimes


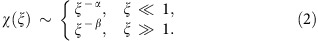


Simulated clone size distributions, displayed in Fig. 4b, confirm the predicted scaling form. In real systems, the scaling form has a range of validity, say between some minimal and maximal frequency *x*_min_ and *x*_max_ that are set by details of the growth processes, which are beyond the scope of our model. For instance, the maximal frequency *x*_max_=*O*(1) accounts for the discrete nature of growth during the first few cell divisions. Similarly, *x*_min_=*O*(1/*N*) may reflect mutations that are born behind the front such that our gene surfing theory does not apply.

The two power-law exponents *α* and *β* are constrained by the scaling behaviour of the mean frequency of mutants, as follows. For a given mutation rate *μ* per cell division, the expected frequency 〈*X*_tot_(*t*)〉 of mutants at the surface of a growing *D*-dimensional sphere satisfies


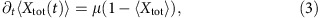


where time is measured in units of generations at the front. Hence, as long as 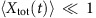, we have 〈*X*_tot_(*t*)〉=*μt*. Every generation, the radius grows by *λ*, such that it takes the population *t*=*R*/*λ* generations to grow to size *N*∝*R*^*D*^. The length *λ* can be interpreted as a measure for the thickness of the growth layer at the edge of the colony in units of the linear dimension of the cells. The mean frequency at radius *r* then is 〈*X*_tot_(*r*)〉=*μr*/*λ*, and the population mean follows as 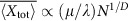. This implies an expected total number of mutants of


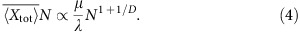


As we ignore cell death, there are on average *μN* mutations occurring during the growth of the population. Hence, the frequency of a single clone is, on average, 
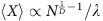
, which constrains the integral over Π(*x*) since 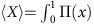. Inserting the scaling relation reported in equation (1), we obtain for large population *N*≫1


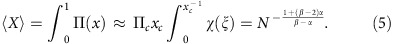


Here, we used that *α*<1 and *β*>1 such that integral over the scaling function *χ*(*ξ*) is finite and dominated by *ξ*-values in the crossover region, *ξ*=*O*(1). Equation (5) can only hold for all *N* if we have the scaling relation


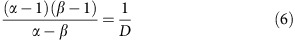


Thus, computing the exponent for either the bubble or the sector regime uniquely determines the exponent of the other regime. Note that in the well-mixed case, *D*→∞, equation (6) correctly reproduces a single power law with *α*=*β*=1. Here, we derive the exponent for the bubble regime in two dimensions; the sector regime and higher dimensions are described in Supplementary Notes 4 and 5.

### Bubble distribution in two dimensions

The area *A*, length *L*_||_ and width *L*_⊥_ of a bubble are determined by the statistical properties of its boundaries, which result in the general relationship 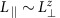 (see Fig. 4a). We can relate the distribution of the area 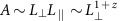 of a bubble to the distribution of the lateral bubble size *L*_⊥_ through


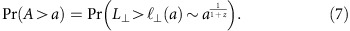


For neutral mutations, we must also have the conditional probability 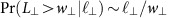 of reaching transverse size *w*_⊥_ given an initial size 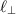, because, asymptotically, each front segment of size 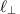 has the same chance of expanding up to size *w*_⊥_. This implies 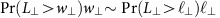. Since the choice of *w*_⊥_ is arbitrary, it must hold that 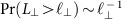 for large enough 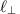. Combining this with equation (7) yields the distribution of clone frequencies


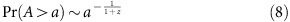


for asymptotically large bubbles, that is, *α*=1/(1+*z*) in two dimensions. In *D* dimensions, *α*=[1+*z*/(*D*−1)]^−1^ (Supplementary Note 5). The scaling relation in equation (6) can be used to find the exponent in the sector regime *β*=[1+*D*(*z*−1)]/[(*D*−1)(*z*−1)]. Numerical values for the exponents in a variety of scenarios are given in Table 1.

### Meta-population model simulations

We simulated range expansions using a meta-population model based on the Eden model^39^: space is divided into a 2D or 3D square grid, whose voxels can be empty, wild type or mutant type. In general, each voxel represents a sub-population, also called deme, consisting of multiple individuals, and the lattice size *a* is a model parameter that characterizes the spatial extent of a deme.

The grid is initialized by filling the central voxel with wild type. In each step, we choose a filled voxel *i* that has at least one empty neighbour and one of its empty neighbouring voxels *j* at random and copy the state of *i* into *j*. If *i* is wild type, we switch (‘mutate') voxel *j* to the mutant type with probability *μ*. A generation corresponds to a number of steps equal to the number of voxels that have at least one empty neighbour at the beginning of the generation.

To accommodate the limited number of observable SNPs in the experimental data when comparing with simulations, we sample *M* mutations with frequency above the minimum experimentally detectable frequency from the simulated distribution. Here, *M* is the number of observed mutations in one experiment (Supplementary Table 1). The sampling is repeated 10,000 times, and the resulting distributions binned across frequencies. The result is shown as the grey area in Fig. 3 containing 95% of the sampled distributions.

To simulate the effect of intermediate antibiotic concentrations on colony growth and spreading of resistant individuals observed in experiments (Fig. 6c), we extended the meta-population model simulation to accommodate sudden changes in the environment and death of the wild type. Environmental changes are modelled by changing the relative growth rate of mutants, *g*_mut_, and wild type, *g*_wt_, at time *T* during the simulations, which corresponds to when the antibiotic is administered. We define the selective advantage/disadvantage *s* of the mutant as *s*=*g*_mut_/*g*_wt_−1. Without death of the wild type, the environmental change would only be felt by the individuals at the front, in contradiction to experiment, where we frequently observe the escape of mutants from the bulk of the population. Therefore, we introduce the possibility of death: after time *T*, any of the *n*_wt_ wild type cells has a chance *δ* of dying each generation. In addition to the standard algorithm above, each generation then incurs *δn*_wt_ additional iterations, in which one wild-type voxel is deleted. More details on the algorithm are reported in Supplementary Methods. To simulate the experimental scenario in Fig. 6, we first grow a population with deleterious mutations until time *T* (*μ*=2 × 10^−4^, *s*=−0.4), whereafter new mutations are not allowed, mutants switch to having a selective advantage (*s*=0.4), and wild-type death is switched on. Supplementary Movie 3 shows the qualitative agreement with the experimental results in identifying an intermediate death rate that minimizes colony growth.

### *E. coli* experiments

For the sequencing experiments, we used a K12 *E. coli* strain MG1655 where the *mutT* gene was replaced by *Cm*_*R*_, conferring resistance to chloramphenicol. This strain has an elevated mutation rate for A/T→C/G transversions over wild-type MG1655 by a factor of about 150 on average^40^. For the trapping experiments in Fig. 6, we used a pair of MG1655 strains, one expressing CFP from a plasmid, the other expressing YFP and a resistance gene to chloramphenicol.

In liquid culture, *E. coli* were grown continuously shaken at 37 °C in Luria-Bertani (LB) broth (10 g l^−1^ tryptone, 5 g l^−1^ yeast extract and 10 g l^−1^ NaCl). For plates, 2% w/v bacto agar was added to the media before autoclaving. Antibiotics were added after autoclaving to cooled media.

To prepare cells of *mutT E. coli* for sequencing, we grew them in liquid culture up to a density of 10^8^ cells per ml and then (i) plated single cells for colonies and (ii) inoculated well-mixed cultures from single cells following a 10^7^ dilution. Parallel tubes (86) were inoculated with 100 μl of the dilution and incubated well shaken for about 10 h (around 30 generations). Cells were harvested in log phase, judged by OD600 (Supplementary Table 1). To grow well-mixed populations on plates, 16 LB plates were inoculated with 100 μl of the dilutions and incubated. Every 90 min, 100 μl PBS was pipetted onto each plate and vigorously spread using glass beads. After ∼20 h, seven plates displayed a uniform bacterial lawn. Two lawns were resuspended via vortexing. Colonies were grown for 3 to 5 days up to a diameter between 1 and 1.5 cm (Supplementary Fig. 2). For five colonies (colonies 1–5 in Supplementary Table 1 and Supplementary Fig. 6), cells were resuspended by vortexing and the genomic DNA extracted for each population. For the last colony (colony 6), the colony was cut in four parts via a glass pipette for the centre portion (IN) and a razor blade (for the remaining outer ring). The outer ring was divided into three parts: 1/8, 1/4 and the remainder of the ring (around 1/2 ring), as shown in Supplementary Fig. 6. The DNA of each portion was extracted separately (details are in Supplementary Table 1). Genomic DNA was extracted from all population following the Epicentre MasterPure DNA Purification Kit Protocol.

Illumina library preparation was performed on each sample and final libraries were used at similar concentrations to achieve similar coverage across samples. The average insert size of the library was ∼200 bps. The sample libraries were then sequenced on the HiSeq 2500 at the QB3 Vincent J. Coates genomic facility at UC Berkeley using 150 paired-end reads. Because the library insert size is on average smaller than 300 bps, the two paired-end reads overlap, providing two independent calls for each base in the overlapping region^41^,^42^. Each sequencing lane accommodated six distinct samples providing, on average, a coverage of 1,000 × per sample. One colony (colony 5 in Supplementary Table 1) was also sequenced separately in one lane, generating a 6,000 × coverage for this sample.

### Processing of sequencing data

The reads of each sample were processed according to the following pipeline. Read quality was assessed via FastQC to check for base sequence quality, GC content bias, length distribution and adaptor contamination. Because some adaptor contamination was determined, reads were filtered for library adaptors and trimmed when necessary.

Paired-end reads were then merged using BBmerge (available at http://sourceforge.net/projects/bbmap), which identifies the optimal relative position of corresponding read pairs and generates a unique consensus read with combined quality scores (on average, 90% of the reads were uniquely merged and used for the subsequent analysis). The resulting sequencing error was on average lower than 10^−6^ for all samples.

BreSeq^43^ was then used to map the merged reads to the reference genome of *E. coli* strain MG1655 (NCBI id: NZ_CP009685.1, ref. 44). SNP-calling and frequency calculation was also performed via BreSeq conditioning on at least four independent read calls.

Genomic regions with unusually high density of SNPs were omitted. To determine whether SNPs were to be disregarded, a sliding window of 5 kbps was passed across the genome counting the number of SNPs with frequency lower than a varying threshold. For each frequency threshold *i*, the average number of SNPs per window *n*_*i*_ was recorded. If a window showed a number of SNPs with *P*-value lower than 0.001 (assuming a Poisson distribution with mean *n*_*i*_), all the SNPs in that window were removed. This procedure takes into account that SNPs with lower frequency are more numerous and thus more densely populate the genome, while providing a more conservative approach than simply removing regions with high SNP density regardless of their frequency. The flagged regions were often shared among samples and annotated as repetitive regions in the reference genome.

Among the remaining putative mutations, SNPs present in more than 40% of the population were deemed mutations carried by the seeding cell(s) and removed. Three of the liquid cultures appeared to have been seeded by multiple cells, since they contained SNPs at frequency equal to 50%. The remaining SNPs with corresponding frequency were then used to generate the clone size distribution in Fig. 1 for all samples, with the exception of colony 6. The details on how we combined the SNPs from the different portions of colony 6 are reported in Supplementary Methods.

### *S. cerevisiae* experiments

For the fluorescent marker experiments in budding yeast (Fig. 2), we used the W303 *S. cerevisiae* strain JRY10643 derived from JRY9628 (ref. 23). This strain employs the Cre-loxP recombination system to switch stochastistically from a red to a green fluorescent state at a rate *μ*=1.6 × 10^−3^ per cell division. *S. cerevisiae* were grown at 30 °C in yeast extract peptone dextrose (YPD) (20 g l^−1^ peptone, 10 g l^−1^ yeast extract and 20 g l^−1^ dextrose). For plates, 2% w/v bacto agar was added to the media before autoclaving. To grow colonies from single cells, saturated overnight culture was diluted 1:10 in fresh media and grown for another 4.5 h. The resulting culture was diluted in PBS to give about 50 cells per ml. A volume of 100 μl of this dilution was spread on YPD plates (containing roughly 20 ml of YPD with 2% agar) that had been dried at room temperature for at least 24 h. After drying, the plates were wrapped with parafilm and incubated at 30 °C for 5 days.

### Image analysis

For the timelapse movie (Supplementary Movie 1), single cells of JRY10643 were inoculated on YPD plates, incubated in a stage-top incubator fitted to a Zeiss AxioZoom microscope and grown overnight. One colony was selected and imaged every 30 min in both the red and the green fluorescent channel. For Fig. 2d, a colony was imaged every 24 h and images overlaid in Adobe Photoshop. To image sectors and bubbles on the single-cell scale, a colony was cut out from the agar plates and imaged on a Zeiss LSM700 confocal microscope, using 488 and 555 nm lasers. A *z*-stack was recorded and later combined by maximum intensity projection.

Colonies were imaged on a Zeiss AxioZoom v16 upright microscope. The red and green fluorescent channel were recorded separately, and exposure times were set automatically by the software for each colony and channel. To measure the mutant clone size distribution in the converting budding yeast strain, we used an automated thresholding algorithm with a locally adaptive threshold. To detect large clones, we removed small object by computing the geodesic opening of the green channel image before binarizing with a locally (50 pixel radius) adaptive threshold. For the detection of small bubbles, we computed the top hat transform of the green channel, using a 15 pixel radius disk as the structuring element, which effectively removes large elements from the image. The resulting image was then segmented using an adaptive threshold in a 15 pixel radius neighbourhood. Finally, the two segmented images were overlaid and eroded by 1 pixel to obtain the final segmentation. In Fig. 2b, we also show the result for no erosion and 2 pixel erosion (grey area).

The imaged budding yeast colonies are not strictly 2D but have a roughly conical shape. Small clones thus occupy stretched 3D volumes 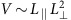, of which only the projection can be observed under the microscope. To take this projection error into account, we consider the volume *V* of bubbles growing in 3D, and, assuming isotropic growth, we compute the projected area 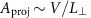, where *L*_⊥_ is the size of the bubble section transverse to the growth direction. Using *z*_3*D*_=1.61, this leads to


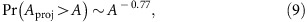


which serves as an upper bound for the case where bubbles are equally extended horizontally as they are vertically. Experimentally, the best fit to the low-frequency regime of the clone size distribution in Fig. 2b gives an exponent of roughly 0.61, which is consistent with bubbles that have a small degree of three-dimensionality but grow mostly in the *x*-*y* plane.

### Data availability

The alignment files obtained from sequencing the *E. coli* populations are available in the Sequence Read Archive (SRA) with access code SRP078606. The rest of the data that support the findings of this study are available from the corresponding author upon request.

## Additional information

**How to cite this article:** Fusco, D. *et al*. Excess of mutational jackpot events in expanding populations revealed by spatial Luria–Delbrück experiments. *Nat. Commun.* 7:12760 doi: 10.1038/ncomms12760 (2016).

## Supplementary Material

Supplementary Information Supplementary Figure 1-7, Supplementary Table 1-3, Supplementary Note 1-5 and Supplementary Methods and Supplementary References

Supplementary Movie 1 Time-lapse movie of an *S. cerevisiae* colony growing from single cell.

Supplementary Movie 2 Eden model simulation growing up to N=500,000 cells with a mutation rate equal to 0.001 (wild-type in purple and mutants in yellow)

Supplementary Movie 3 Simulations showing the effect of antibiotic on the growth of colonies containing resistant bubbles (yellow) for three death rates. Intermediate death rates show minimal growth and containment of resistance, while high death rates result in a large entirely resistant population

## Figures and Tables

**Table 1:** Predictions for the asymptotic behaviour of the clone size distribution.

Scenario	*z*	Low frequency	High frequency
Well-mixed	0	*x*^−1^	*x*^−1^
2D flat front	2	*x*^−1/3^	*x*^−3^
3D flat front	2	*x*^−1/2^	*x*^−2^
2D rough front*	3/2	*x*^−2/5^	*x*^−4^
3D rough front	1.61	*x*^−0.55^	*x*^−2.3^

The dynamical exponent *z* summarizes the statistical properties of the sector and bubble boundaries. The low-frequency regime corresponds to bubbles, the high-frequency regime to sectors. The well-mixed scenario, where bubbles and sectors are not distinct, corresponds to *D*=∞ and is characterized by a single power-law regime. The experimental scenario presented here is well described by the case of 2D rough fronts (*), where *z*=3/2 is given by KPZ (Kardar–Parisi–Zhang) interface growth dynamics^30^.
