# Supplementary material for: Excess of mutational jackpot events in expanding populations revealed by spatial Luria–Delbrück experiments
Source: Nat Commun. 2016 Oct 3;7:12760. doi: 10.1038/ncomms12760 (PMC5059437; doi:10.1038/ncomms12760)
Supplement: Supplementary Information — Supplementary Figure 1-7, Supplementary Table 1-3, Supplementary Note 1-5 and Supplementary Methods and Supplementary References [file ncomms12760-s1.pdf]

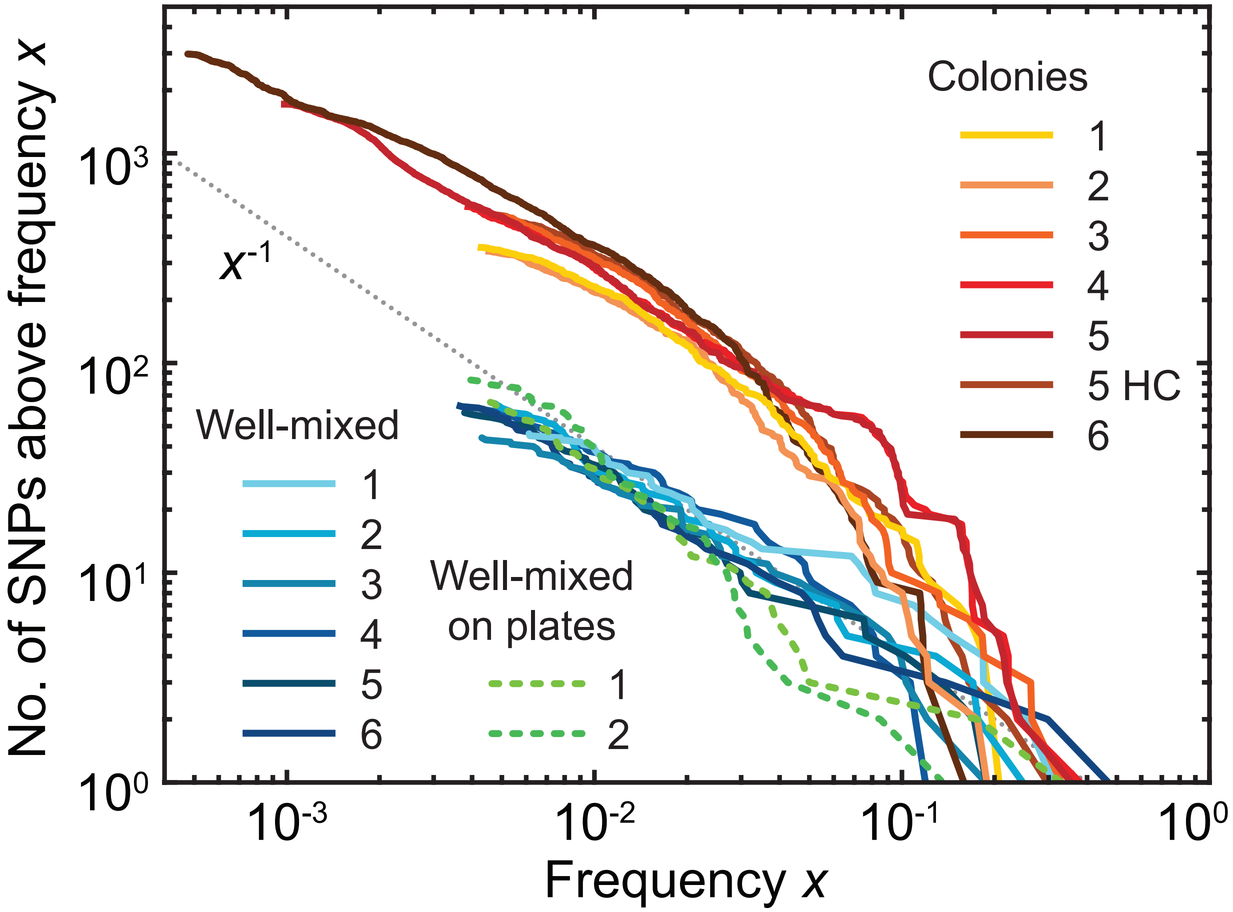

Supplementary Figure 1 | Clone size distribution obtained from sequencing populations of *E. coli*. Populations were grown either as colonies (warm colors), in well-mixed liquid culture (cool colors), or well-mixed on agar plates (green dashed). Same data as in Fig. 1 in the main text, with legend added. Details on the populations are reported in Supplementary Table 1.

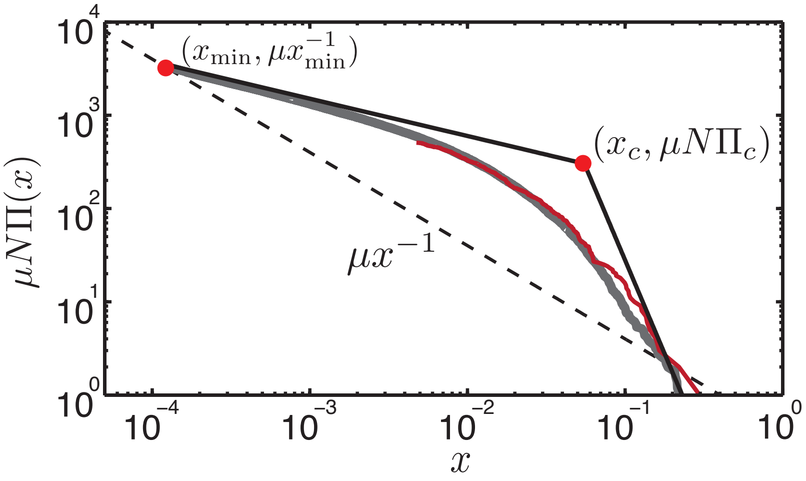

Supplementary Figure 2 | Visualization of the fitting procedure. Visualization of the fitting parameters of the clone size distribution obtained by sequencing colony 1. The crossover position $(x_c, \mu N \Pi_c)$ is found by minimizing the square deviation between the data (red) and the Eden model simulation results (gray). We find that the bubble regime intersects the well-mixed expectation (dashed black line) at frequency $x_{\min}$ below which we assume a well-mixed behavior. The same procedure is repeated for all colonies, generating the parameters in Table 3.

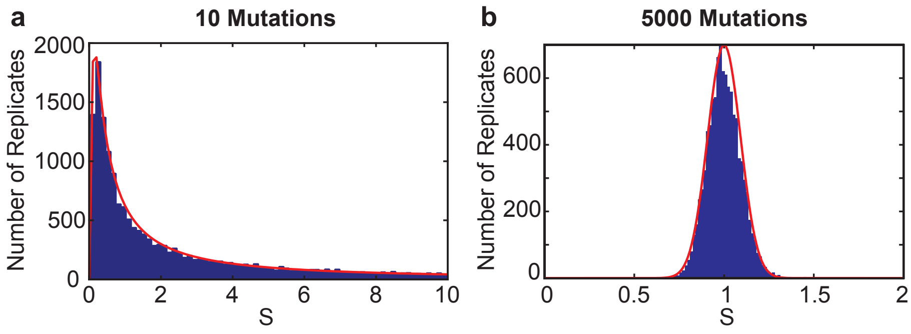

Supplementary Figure 3 | Shape of mutant number distributions for different number of mutations. Histograms of the total number of mutants X_{tot} over the typical total number of mutants $\langle X \rangle_{\mu N}$ ($S = X_{\text{tot}} / \langle X \rangle_{\mu N}$) for a number of mutations much lower than $1/\Pi_c \approx 50$ (10 mutations on the left) and much higher than $1/\Pi_c$ (5000 mutations on the right). In (**a**), large events are rarely sampled and the histogram is well fitted by a Levy stable distribution $L_{2/5,1}$ (red line). The mode of the distribution (typical total number of mutants) is much smaller than its mean (typical is 0.0016, mean is 0.011). In the second case (**b**), the central limit theorem applies and the sample is well approximated by a Gaussian distribution (red line, typical is 5.5, mean is 5.6). The histograms were obtained by sampling 20,000 replicates from the empirical clone size distribution obtained from simulations of colonies of size $N = 2 \times 10^5$.

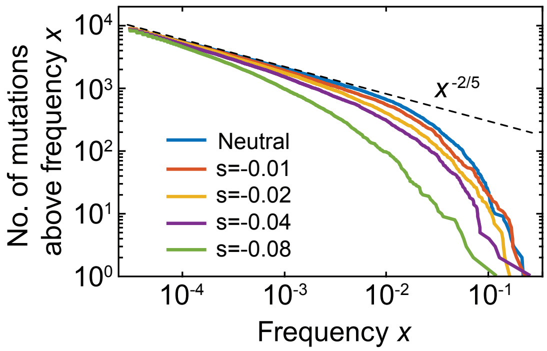

Supplementary Figure 4 | Clone size distribution for deleterious mutations. The formation of sectors and large bubbles is suppressed for mutations with a selective disadvantage *s*. The power-law corresponding to bubbles, however, keeps the neutral exponent even for deleterious mutants.

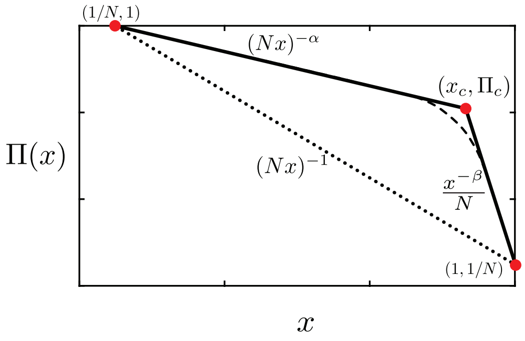

Supplementary Figure 5 | Schematic of the double-log plot of the site frequency spectrum in arbitrary dimension. The dotted line represents the Luria-Delbrück expectation for a well-mixed population of size N. The solid line shows the asymptotic power-law behavior of a range expansion in a population of same size and the dashed line the cross-over in between. The red circles indicate the key points of the distribution (Methods).

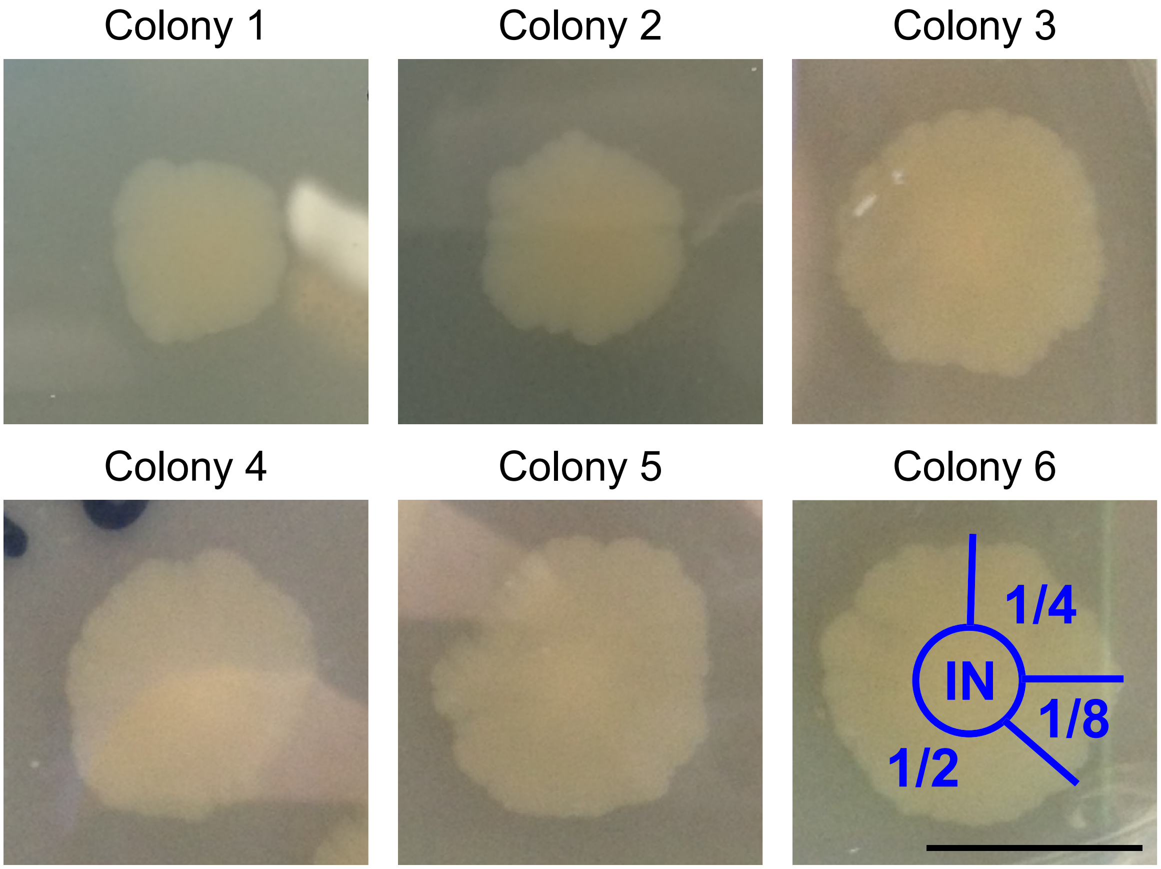

Supplementary Figure 6 | Colonies of the mutator strain *E. coli mutT* used for sequencing. The scale bar corresponds to 1cm. Colony 6 was partitioned in four parts that were sequenced separately: the center (IN), and approximately one eighth (1/8), one fourth (1/4), and one half (1/2) of the outer ring.

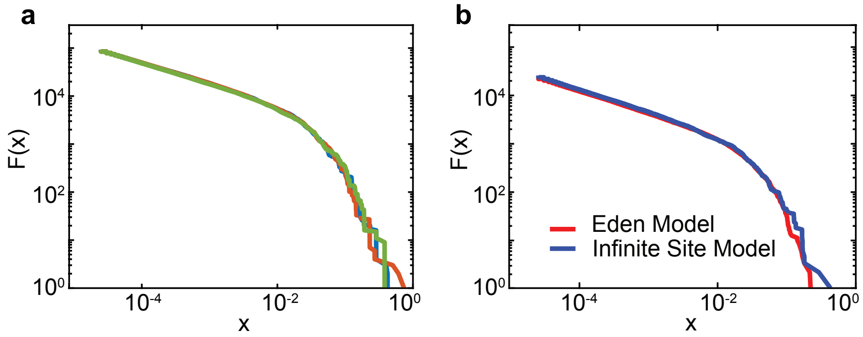

Supplementary Figure 7 | Variation on the clone size distribution generated by tree idiosyncrasies. (**a**) Clone size distribution for three different trees generated using the Eden model and a population size of 2×10^5. (**b**) Clone size distribution generated by the standard Eden model and the Infinite Site Model on the same tree with mutation rate $\mu = 3 \times 10^{-4}$.

	OD 600	Pop. Size ($\times 10^9$)	Mean Coverage	Min. Freq. ($\times 10^{-3}$)	No. of SNPs
Liquid wm 1	0.23	1.3	495	6.13	46
Liquid wm 2	0.25	1.3	836	4.75	64
Liquid wm 3	0.35	1.8	736	4.30	44
Liquid wm 4	0.6	3.0	722	4.29	77
Liquid wm 5	1.26	8.5	954	3.57	62
Liquid wm 6	1.5	9.5	818	3.78	58
Plated wm 1	0.53	>100	857	3.96	83
Plated wm 2	0.66	>100	864	4.59	64
Colony 1	0.13	1.3	887	4.17	357
Colony 2	0.13	1.3	918	4.42	342
Colony 3	0.28	2.8	893	4.81	505
Colony 4	0.36	3.6	884	4.74	508
Colony 5	0.71	7.1	1214	3.79	559
Colony 5 HC	0.71	7.1	6025	0.96	1716
Colony 6: in	0.37	3.7	1348	1.39	222
Colony 6: 1/8	0.08	0.8	964	0.40	474
Colony 6: 1/4	0.15	1.5	574	1.30	384
Colony 6: 1/2	0.32	3.2	1271	1.28	820

Supplementary Table 1 | Summary of the sequenced populations. Liquid well-mixed populations (*"liquid wm"*) were grown in 5ml of LB for about 10 hours, well-mixed populations on plates (*"plated wm"*) were grown for about 20 hours and resuspended in 20ml of PBS (OD from 1:8 dilution of resuspension), while colonies were grown for 3 to 5 days and the OD600 measured from a 1:10 dilution. Populations of the same type are sorted according to their final population size. Biological replicates, i.e., populations inoculated from the same overnight culture, are grouped as follows: (1) Liquid wm 2, 3, 4 and colonies 3, 4, 5; (2) Liquid wm 1 and colony 6; (3) Liquid wm 5, plated wm 1, and colony 1; (4) Liquid wm 6, plated wm 2, and colony 2. The number of cells used to extract DNA for each sample, the mean sample coverage, the minimum observed frequency, and the number of observed SNPs are also reported. Colony 5 was sequenced twice, with the regular, and with additional coverage (Colony 5 HC). Colony 6 was cut into parts that were sequenced separately (Methods in main text and Supplementary Figure 6); the minimum observed frequency reported here is rescaled proportionally to the portion size (Supplementary Methods).

	Liquid Culture	Colonies
**Average Population Size** $\pm$ **s.d.** $(\times \mathbf{10^9})$	1.5 ± 0.2	2.7 ± 0.5
**Empty Plates/Total Plates**	66/86	19/42
μ **[95% C.I.]** $(\times \mathbf{10^{-10}})$	1.8 [1.1, 2.7]	2.9 [1.8, 4.5]

Supplementary Table 2 | Spontaneous mutation rate to spectinomycin in *mutT*. The values were obtained from Luria-Delbrück fluctuation tests in liquid cultures harvested in stationary phase grown in a 96 well plate, and colonies grown for 3 days. The average population size and its standard deviation are computed by counting CFUs of appropriate dilutions, and the confidence interval on the mutation rate is calculated using the binomial test implemented in R.

Colony	x_c	$\mu N \Pi_c$	$x_{\min} (\times 10^{-4})$	$N_{\min} (\times 10^5)$
1	0.067	173	2.8	3.6
2	0.069	154	3.3	4.3
3	0.061	250	1.6	4.5
4	0.059	273	1.4	5.0
5	0.058	311	1.1	7.8
6	0.054	406	0.7	6.4

Supplementary Table 3 | Fitting parameters used to rescale the clone size distributions of the sequenced colonies. When written as $F(x) = \mu N \Pi(x) = \mu N \Pi_c \chi(x/x_c)$, the clone size distributions collapse on a single master curve (Fig. 3 and Supplementary Fig. 2). The minimum frequency $x_{\min}$ below which the bubble regime prediction is expected to fail and its corresponding population size $N_{\min} = x_{\min} N$ are also reported.

Supplementary Note 1: Distribution of mutant numbers in the many-mutations regime (one-step mutations)

Ignoring any cell death and assuming that mutations occur randomly during cell division, one expects that the total number of mutations in a population of size N is sampled from a Poisson distribution with mean μN. Note that because N cell divisions are needed regardless of the spatial structure of the growth process, the total number of mutations is insensitive to the particular demographic scenario. However, the total mutant number will depend on the growth process through the clone size distribution. The mathematical problem of finding the mutant number distribution is complicated in general, and we will not attempt to solve it fully and rigorously. We will, however, give an approximate description of the behavior of the mutant number distribution.

Crucially, we want to assume that the average number $\mu N \gg 1$ of mutations is large, such that its distribution is strongly peaked around the mean and we may simply assume that μN clones arise. The key task, then, is to determine the distribution of the sum of μN random numbers drawn from our above determined clone size distribution.

Even with μN being large, this task is not straightforward because the clone size distribution is broad. The standard central limit theorem does not apply, so long as the largest clones are sampled from the low frequency power law tail with diverging variance. In this case, we expect convergence to the appropriate Levy-stable distribution for large μN [1]. On the other hand, if so many mutations occur that the largest clones are in the sector regime, with a steeply decaying power law, we can apply the standard central limit theorem. In order to determine which limit theorem applies for a given number of mutations and population size, it is first necessary to determine the expected size of the largest clone.

Frequency distribution of the largest clone The probability that the largest clone has frequency lower than y is given by the probability that all μN clones have frequency lower than y, so that

$$\Pr(X_\star < y) = [1-\Pi(y)]^{\mu N}\,, \tag{1.1}$$

where $\Pi(y)$ is the probability that a clone has frequency y or larger. For large μN, we can approximate

$$\Pr(X_\star < y) \approx e^{-\mu N \Pi(y)}, \tag{1.2}$$

The mean frequency of the largest out of μN clones is given by

$$\langle X_\star \rangle_{\mu N} = N^{-1} + \int_{1/N}^{1} \left(1 - e^{-\mu N \Pi(y)}\right) dy\,, \tag{1.3}$$

where the index μN is a reminder for the number of clones sampled. For our analysis, it is not necessary to evaluate this integral exactly, which for many cases is difficult. Note that for $\mu N \Pi(1) \gg 1$, the integral is dominated by values $y < y_m$ with $\mu N \Pi(y_m) = 1$ (otherwise, all values $y < 1$ matter such that the biggest clone is of order 1). To get the overall behavior of the integral, we may estimate [2]

$$\Pi(\langle X_\star \rangle_{\mu N}) \approx \frac{1}{\mu N}\,. \tag{1.4}$$

In a growing well-mixed population, we have $\Pi(x) = (Nx)^{-1}$ and, hence,

$$\langle X_\star \rangle_{\mu N} \approx \mu \tag{1.5}$$

In a range expansion, on the other hand, we have to distinguish two regimes, depending on the magnitude of y_m compared to the cross-over frequency x_c from bubbles to sectors. In two dimensions, we find

$$\langle X_\star \rangle_{\mu N} \sim \begin{cases} (\mu N)^{5/2}/N, & \mu^{1/4} \ll x_c, \\ \mu^{1/4}, & \mu^{1/4} \gg x_c. \end{cases} \tag{1.6}$$

Sampling distributions In the following, we apply the generalized central limit theorem [1] to determine the sampling distributions in the limit of having a large number μN of mutations. To correctly formulate the various scaling limits, we need to first understand how the typical mutant numbers depend on the key parameters of the problem, the mutation rate μ, population size N and the cross-over frequency x_c between bubbles and sectors. Although we know the mean number of mutants (Methods), it is dominated by rare events and thus can be very different from the mode or median of the distribution.

When sampling μN clones, one typically finds clones up to the largest size $\langle X_\star \rangle_{\mu N}$, determined above. Clones even larger than $\langle X_\star \rangle_{\mu N}$ are *typically* not observed (even though they may control the *mean* mutant number). In an ensemble of μN draws from the clone size distribution, the *apparent* mutant number distribution therefore equals $\Pi(x)$ for $x \ll \langle X_\star \rangle_{\mu N}$, but vanishes for $x \gg \langle X_\star \rangle_{\mu N}$ [2].

Given this truncated clone size distribution, we can apply the standard central limit theorem to estimate the typical frequency $\langle X_{\text{tot}} \rangle_{\mu N}$ of mutants as

$$\langle X_{\text{tot}} \rangle_{\mu N} \approx \mu N \int_0^{\langle X_\star \rangle} \Pi(x). \tag{1.7}$$

The resulting $\langle X_{\text{tot}} \rangle_{\mu N}$ will give an estimate of the total mutant number in the vast majority of cases, ignoring the rare big events that actually push up the mean of the distribution. In particular, the dependence of $\langle X_{\text{tot}} \rangle_{\mu N}$ on the mutation rate and population size are key to be able to identify the limiting distribution for large μN. For instance, in a uniformly growing, well-mixed population, we have $\Pi(x) = (Nx)^{-1}$ and $y_m = \mu N/N = \mu$, and thus

$$\langle X_{\text{tot}} \rangle_{\mu N} \approx \mu N \int_{1/N}^{\mu} \frac{dx}{Nx} = \mu \ln(\mu N). \tag{1.8}$$

Note that the typical number $\langle X_{\text{tot}} \rangle_{\mu N}$ of mutants is smaller than the actual mean $\langle X_{\text{tot}} \rangle = \mu \ln(N)$ – this effect will turn out to be even more pronounced in the range expansion case. The typical frequency of mutants allows us to properly scale the mutant distribution: The random variable $X_{\text{tot}}/\langle X \rangle_{\mu N}$ is distributed according to the Landau distribution (up to a numerical scale factor) in the limit $\mu N \to \infty$, while $\mu \ln \mu N$ is fixed [3]. The Landau distribution is the Levy stable distribution $L_{1,1}$, where $L_{\mu,\beta}$ is the Levy stable distribution of order μ and asymmetry parameter β [2].

A different behavior is obtained in the case of a range expansion. Again, we have to distinguish two regimes: When $X_\star \gg x_c$, the integral in Eq. (1.7) is not dominated by the upper bound implying that the typical number of mutants is very close to the mean, $\langle X_{\text{tot}} \rangle_{\mu N} \approx \langle X_{\text{tot}} \rangle \sim \mu N^{1/2}/\lambda$ (from Eq. (4) in the Methods). The distribution around the mean will become Gaussian, because the assumptions of the standard central limit theorem apply.

On the other hand, for low mutation rates such that $X_\star \ll x_c$, or $\mu^{3/2} N \ll 1$, one expects the population not to sample any sectors, which generate frequencies larger than x_c. In the integral in Eq. (1.4), we can then use the pure bubble spectrum $\Pi(x) \sim (Nx)^{-2/5}$ up to N-independent pre-factors. Hence, we obtain

$$\langle X_{\text{tot}} \rangle_{\mu N} \approx \mu N \int_{1/N}^{(\mu N)^{5/2}/N} dx (Nx)^{-2/5} = \mu^{5/2} N^{3/2}. \tag{1.9}$$

In this regime, we have $\langle X_{\rm tot}\rangle_{\mu N} \ll \langle X_{\rm tot}\rangle \sim \mu N^{1/2}$, i.e., the typical total mutant number is much smaller than its mean, which is dominated by exceedingly rare events. Because mean and variance diverge for the power laws corresponding to the bubble regime ($\Pi(x) \sim x^{-\alpha}$ with $\alpha > 1$), we have to apply the generalized central limit theorem [1]. In the two-dimensional case, we then predict that the scaled random variable $S = X_{\rm tot}/\langle X_{\rm tot}\rangle_{\mu N}$ is distributed according to the Levy stable distribution $L_{2/5,1}(s)$ (up to a numerical scale factor) in the limit $\mu N \to \infty$ while $\mu^{5/2}N^{3/2}$ fixed.

The simulated sampling distributions in Supplementary Fig. 3 are indeed consistent with the above asymptotic predictions, confirming in particular the scaling of the typical number of mutations.

Supplementary Note 2: Emergence of double mutants and other complex genotypes

We argue in the main text that the excess of high-frequency mutations can promote the likelihood of rare evolutionary events in range expansion. Here, we make these arguments more precise. To be specific, we first seek to compute the probability that the final population harbors a double mutant AB in both the colony and the well-mixed case. The mutation rate for the first mutation A and the second mutation B shall be μ_A and μ_B, respectively. For simplicity, we restrict the following discussion to two dimensions.

The probability that no B mutation occurs within an A–clone of frequency x is given by

$$\text{Pr(No Double Mutant}|x,N) = e^{-\mu_B x N}, \tag{2.1}$$

in terms of the mutation rate μ_B for the second mutation. By integrating over the site frequency spectrum, we obtain the total probability that a single A clone does not contain a double mutant,

$$\begin{aligned} \text{Pr(No Double Mutant}|\text{Single } A\text{-Clone}) &= \int dx\, \pi(x)\, \text{Pr(No Double Mutant}|x,N) && (2.2)\\ &= 1 - \mu_B N \int dx \Pi(x) e^{-\mu_B x N}, && (2.3) \end{aligned}$$

where $\Pi(x) = \int_x^1 \pi(x)$ is the probability that a clone generated from one mutation has frequency larger than x (Methods). If we treat the number of single mutations as a large deterministic number, $\mu_A N \gg 1$ (as in Supplementary Note 1), we have

$$\text{Pr(No Double Mutant)} = \text{Pr(No Double Mutant}|\text{Single } A\text{-Clone})^{\mu_A N}\,, \tag{2.4}$$

Then, the probability of a double mutant can be estimated as

$$\begin{aligned} \text{Pr(At Least One Double Mutant)} &= 1 - \text{Pr(No Double Mutant)} && (2.5)\\ &\approx \mu_A \mu_B N^2 \int dx \Pi(x) e^{-\mu_B x N}, && (2.6) \end{aligned}$$

where we have approximated

$$\begin{aligned} \text{Pr(No Double Mutant}|\text{Single } A\text{-Clone})^{\mu_A N} &= \left(1 - \mu_B N \int dx \Pi(x) e^{-\mu_B x N}\right)^{\mu_A N} && (2.7)\\ &\approx 1 - \mu_A \mu_B N^2 \int dx \Pi(x) e^{-\mu_B x N}\,, && (2.8) \end{aligned}$$

which is valid if the total probability for a double mutant is small.

One can show that Eq. (2.8) is well-approximated by truncating the integral such that

$$\Pr(\text{At Least One Double Mutant}) \approx \mu_A \mu_B N^2 \int_{1/N}^{(\mu_B N)^{-1}} dx \Pi(x). \tag{2.9}$$

In the well-mixed case, where $\Pi(x) = (Nx)^{-1}$, we obtain

$$\Pr(\text{At Least One Double Mutant})_{\text{w.m.}} = -\mu_A N \mu_B \log \mu_B. \tag{2.10}$$

for $\mu_B N > 1$. When the B-mutation rate is so small that $\mu_B N < 1$, the probability for a mutant crosses over to μ_B times the mean number of single mutants $\mu_A N \log N$.

In range expansions, still more regimes need to be distinguished. When $x_{\min} > (\mu_B N)^{-1} > x_c$, the distribution of bubbles matters and the double mutant probability scales as

$$\Pr(\text{At Least One Double Mutant})_{\text{r.e.}} \sim \mu_A N \mu_B^{2/5}, \tag{2.11}$$

with a pre-factor that depends on the frequency $x_{\min}$ above which the power law for bubbles holds (Methods). Finally, one observes a crossover to μ_B times the mean number of A-mutants, $\sim \mu_A N^{1/2}$, when $(\mu_B N)^{-1} > x_c$ so that the single mutants numbers are closely centered at the mean (Supplementary Note 1).

In Fig. 5b in the main text, we plotted the ratio $\sim \mu_B^{-3/5}$ of double mutant probabilities in the range expansion case, Eq. (2.11), and in the well-mixed case, Eq. (2.10).

Note that our discussion is not specific to the emergence of double mutants. We could interpret μ_B as the probability that a drug resistant mutant survives a downstream dilution process. The "double mutant" event would then be the event of at least one resistant mutation surviving dilution.

Alternatively, we could interpret μ_B as the probability of getting k additional mutations given the first mutation has occurred. This would then lead to a model of how jackpot events predispose to getting $k+1$ driver mutations, which could be relevant in the context of cancer risks [4].

Supplementary Note 3: Impact of deleterious mutations

As mentioned in the main text, mutations that confer resistance often come at a cost, which can influence their distribution of clone sizes. To emulate costly resistance mutations, we simulated a range expansions in which all incoming mutations carry a growth rate disadvantage. We find that the probability of forming a sector quickly goes to zero as the selective disadvantage increases (Fig. 6a in the main text); even for relatively mild disadvantages of a few per cent beginning sectors die off quickly and mutants are trapped inside the bulk. This has implications for the successful treatment of such populations, as discussed in the main text and in Fig. 6.

The clone size distribution of deleterious mutants obtained from Eden simulations (Supplementary Fig. 4) shows that, while small clones are generated at an almost neutral rate, large clones are strongly suppressed compared to the neutral case. The dependence of the cross-over frequency of the largest "neutral" clones on the selective disadvantage s can be estimated as follows. Selection will bias the transverse size $\ell_\perp$ of clones to shrink in time with velocity $v_\perp$, which is proportional to $\sqrt{s}$ for small $s \ll 1$ [5]. Selection and genetic drift will be of comparable strength when the deterministic "time" $\ell_\perp / v_\perp$ to shrink to 0 is comparable to the corresponding stochastic time scale $\ell_\perp^z$ (with z being the dynamic exponent introduced in the main text). Hence, we estimate the cross-over from neutral to selection-dominated to occur at a transverse scale of $\ell_{\perp,s} \sim v_\perp^{1/(1-z)}$. Since transverse scale and area are related by $\ell_\perp^{1+z} \sim a$ and area is proportional to frequency, we obtain the cross-over frequency

$$x_s \sim v_\perp^{\frac{1+z}{1-z}} \sim s^{\frac{1+z}{2(1-z)}}. \tag{3.1}$$

which corresponds to $x_s \sim s^{-5/2}$ for $z_{2D} = 3/2$.

Supplementary Note 4: Alternative Derivation of Sector Size Distribution

Although the scaling relation relating the bubble and sector regime power law exponents, Eq. (6) in the main text, uniquely determines the exponent β of the sector regime, we can understand the asymptotic power law at high frequencies from first principles, as follows.

Clones that establish generate sectors that are bounded by two random walks diffusing in angle space with a radius-dependent diffusion constant $D_\Phi(r) \sim D_X/r^2$ [5]. If a sector arises at radius r, the asymptotic mean square angle will scale as

$$\langle \alpha_r^2 \rangle \sim r^{2(\zeta-1)}, \tag{4.1}$$

where $\zeta = 1/z$. For sufficiently large populations (such that further boundary meandering is negligible compared to the width of the clone), the clone frequency $x(r)$ of a mutation arising at r is $x(r) \sim \alpha_r/C_{D-1}$, where $C_{D-1} = 2\pi^D/\Gamma(D/2)$ is the surface area of a (D-1)-dimensional sphere of unit radius. This means that to reach a frequency x, a mutation has to arise at the latest at

$$r(x) \sim x^{\frac{1}{(D-1)(\zeta-1)}}. \tag{4.2}$$

The probability $u(r)$ of such mutation to form a sector can be computed by invoking the neutrality assumption: A sector that achieves asymptotic angle α_r stems from a single individual present at radius r among the approximately $(\alpha_r r)^{D-1}$ that could have equally likely established. It follows that

$$u(r) \sim \frac{1}{(\alpha_r r)^{D-1}} \sim r^{-\zeta(D-1)}. \tag{4.3}$$

The total number of sectors N_{sec} with frequency greater than $x(r)$ must have arisen from a core of radius r and can be computed by integrating the probability μr of mutations to occur at radius r times its establishment probability $u(r)$,

$$N_{\text{sec}}(R) \sim \mu \int_0^R dr r^{D-1} u(r) \sim R^{-\zeta(D-1)+D}, \tag{4.4}$$

where μ is the mutation rate.

The probability $P(X > x)$ of a sector having frequency larger than x is therefore

$$P(X > x) \sim \frac{N_{\text{sec}}(R(x))}{N_{\text{sec}}(R_{\text{f}})} \sim \left(\frac{x}{x_{\text{min}}}\right)^{\frac{1}{(D-1)(\zeta-1)}-1}, \tag{4.5}$$

where R_{f} is the final colony radius and x_{min} is the corresponding minimum frequency cutoff. This exponent is consistent with the value predicted by the scaling relation, eq. (6) in the main text. Table 1 in the main text summarizes the exponents α and β for the diffusive and super-diffusive sector boundaries in 2 and 3 dimensions.

Supplementary Note 5: Bubbles in D Dimensions

We assume that, in D dimensions, the volume of a bubble is D dimensional and can be estimated by $V \sim L_\perp^{D-1} L_\parallel$. (This assumption could be problematic if bubbles have complicated fractal dimensions.) We are then interested in predicting the distribution of the bubble volume $V \sim L_\perp^{D-1} L_\parallel \sim A_\perp^{1+z/(D-1)}$, which can be

expressed in terms of the bubble area $A_\perp \equiv L_\perp^{D-1}$ transverse to the expansion direction. In the neutral case, we can argue $\Pr(A_\perp > a_\perp) \sim a_\perp^{-1}$, analogously to the $2D--$case discussed in the main text, to find

$$\Pr(V > v) = \Pr(A_\perp > a_\perp(v)) \tag{5.1}$$

$$\sim a_\perp^{-1}(v) \sim v^{\frac{1}{1+\frac{z}{D-1}}} = v^{-\alpha} . \tag{5.2}$$

Since, the second exponents β follows from our scaling relation Eq. (6), we predict

$$\alpha = \left[1+\frac{z}{D-1}\right]^{-1} \tag{5.3}$$

$$\beta = \frac{1+D(z-1)}{1-z+D(z-1)} . \tag{5.4}$$

With $z_{3D} \approx 1.61$, these relations predict $\alpha_{3D} \approx 0.55$ and $\beta_{3D} \approx 2.32$, see also Table 1 in the main text. Our three dimensional Eden model simulations are consistent with these predictions (Fig. 4b in the main text).

Supplementary Methods

Estimates of mutation rate in liquid culture and colonies. To verify that the mutation rate is independent of the mode of growth, we performed Luria-Delbrück experiments using *mutT* populations grown as colonies from single cell harvested after three days, and overnight liquid cultures. The liquid cultures were inoculated with 2 to 10 cells. All populations were grown up to similar size and the precise size was measured by plating dilutions on non-selective plates. The mutation rate was estimated from the fraction of populations that exhibited no growth two days after being moved onto plates with 60μg/ml of spectinomycin. The reported spontaneous mutation rate to spectinomycin in K12 *E. coli* is about 2×10^{-10}, generated by an aminoacid substitution in a ribosome unit [6]. Because the required mutation consists in a C/G to T/A transversion [7], whose rate is not affected by the *mutT* deletion of the strain, we expect similar mutation rates as in the wild-type. Supplementary Table 2 shows that both growth modes generate similar mutation rates. This supports our assertion that the increased number of mutants in colonies reported in Fig. 1 cannot be explained by a different mutation rate.

SNP merging in composite colony 6. For colony 6, whose different portions were sequenced separately, the clone size distribution was determined by combining the filtered SNPs from the distinct colony portions (all the fixed SNPs were fixed in all portions and thus removed). First, each SNP total population frequency x_t corresponds to

$$x_t = \frac{\sum_i \rho_i x_i}{\sum_i \rho_i}, \tag{5.5}$$

where the sum runs over the different colony portions that contain the SNP, ρ_i is the OD 600 density of portion i and x_i is the frequency of the SNP in portion i.

Secondly, we accounted for the higher resolution of portion 1/8. For each colony sample i, we defined the minimum observable frequency $\xi_i = \frac{\rho_i \min(x_i)}{\sum_i \rho_i}$, where $\min(x_i)$ is the minimum SNP frequency observed in sample i. Because of the partitioning of the colony, these frequencies satisfy the relation $\xi_{\text{in}} \sim \xi_{1/2} > \xi_{1/4} > \xi_{1/8}$ (see Supplementary Table 1). We then identified all the SNPs in portion 1/8 that had rescaled frequencies lower than ξ_i, where i corresponds to each of the larger three portions. Assuming that the frequency distribution of these SNPs is homogenous across the colony, we can extend their presence to the other samples, by adding $\rho_i/\rho_{1/8}$ inferred mutations to the list of mutations in portion i with frequency equal to the rescaled frequency in portion 1/8. Finally, the merged list of mutations for each portion is used to calculate the clone size distribution of colony 6 in Fig. 1 and Supplementary Fig. 1.

Sequencing control using the wild type. To determine whether the number of SNPs detected was consistent with previously reported mutation rates, we grew and sequenced a well-mixed population and a colony of the wild type strain *E. coli* MG1655 with a functional *mutT* gene following the same protocol we used for the mutator strain. In particular, the colony was sequenced in partitions similarly to colony 6 in Supplementary Fig. 6. After analysis of the results, we detect only a single SNP in the well-mixed population, whose minimum detectable frequency was around 3×10^{-3}. By comparing the number of SNPs above this frequency observed in the mutator strain, we infer that the mutator strain is characterized by an approximately 100-fold increase in the mutation rate. For the colony, we detect a total of 23 SNPs combined across the different regions above frequency 3×10^{-3} compared to the 1000 SNPs found in colony 4, which suggests a 50-fold increase in mutation rate. The reported mutation rate in wild type *E. coli* is around 0.001 per genome per generation [8]. The expected mutation rate in our *mutT* deletion strain is thus roughly 0.1 per genome per generation. This number is consistent with the mutation rate inferred from fitting the well-mixed clone size

distribution in Fig. 1 to the standard Luria-Delbrück expectation, which gives ≈ 0.4. Moreover, although the number of detected SNPs in the wild type is not sufficiently large to accurately compute the clone size distribution, it supports the observation that colonies produce approximately 10 times more mutations with clones above frequency 3×10^{-3} compared to equally large well-mixed populations.

Correction of clone size distribution at low frequencies. Even if sequencing errors can be neglected, the true and the observed frequency of a SNP can still differ because only a subsample of reads are sequenced at the SNP position. Although this is an issue across all frequencies, the relative error is much larger a low frequencies, where one extra read can have a strong impact on the reported frequency. Assuming that sampling is the only source of noise, the variance associated with a SNP with frequency x is $v(x) = x(1-x)/n$ where n is the coverage at that position [9]. The relative error on the frequency scales as $x^{-1/2}$ and is especially relevant at low frequencies. The (integrated) clone size distribution $F(x)$ can then be better approximated by

$$F(x) = \sum_i \int_x^1 \mathscr{B}[nx', nx_i] dx', \tag{5.6}$$

where the sum runs through all SNPs and $\mathscr{B}(y', y)$ is a binomial distribution function with mean y. The clone size distributions plotted in Fig. 1 and rescaled in Fig. 3 were obtained in this way.

Tree-conditioned clone size distribution and Infinite Sites Model. Since the clone size distribution in simulations, sequenced populations, and imaged colonies is derived from mutations that occur on one or few genealogical trees (one for sequencing, few hundreds for imaging, and few thousands for simulations), the size of the different clones are not independent, but are constrained to lie on a given tree. The individual characteristics of one specific tree can affect multiple clones and may thus give rise to a clone size distribution that is significantly different from one generated via another tree.

To test to what extent tree conditioning generates variation in the clone size distribution, we used the Eden model to generate three colonies of identical size and analyzed the complete clone size distribution. Supplementary Figure 7a shows that the inferred clone size distributions for the simulated trees show some variation in the sector regime, but overlap well in the bubble regime. This is in agreement with the intuition that small clones are less affected by the specific shape of the tree compared to large clones. Although tree idiosyncrasies generate some variation in the clone size distribution, the resulting noise is by far lower than that generated by subsampling (Methods), which is accounted for via bootstrapping in Fig. 3.

Conditioning on the tree, we also show that the results from the standard Eden model simulations, in which mutants cannot mutate further, are consistent with an Infinite Site Model implemented on top of the Eden model, in which they can. Supplementary Fig. 7b shows the clone size distribution on the same tree using the standard Eden model and an infinite site model. Also in this case, the resulting variation at high frequencies is less than what is generated by subsampling.

Dynamical simulations for the effect of antibiotics on colony growth. To simulate the effect of intermediate antibiotic concentrations on colony growth and spreading of resistant individuals observed in experiments (see Fig. 6 in the main text), we adapted the Eden model simulation to accommodate sudden changes in the environment and death of the wild type. Environmental changes are modeled by changing the relative growth rate of mutants and wild type before and after a certain time T during the simulations, and by introducing a wild-type death rate δ after time T, which marks the administration of the antibiotic.

The algorithm follows the following steps for each generations:

1. Count the number of cells n with at least one empty neighbor.

2. Count the total number of wild type cells n_{wt}.
3. Define $R = n$ reproductive steps and $D = d \cdot n_{\mathrm{wt}}$ death steps.
4. During any of the $R + D$ steps,
    (a) Pick a random integer u in the $[0, R+D)$ interval.
    (b) If $u < R$ perform a reproductive step as in the standard Eden model,
    (c) Otherwise, pick a random wild type cell and delete it.

In each generation, the algorithm ensures an average number of R births and D deaths, as desired.

Fitting of the clone size distribution from sequencing data. To estimate actual values from the empirical clone size distribution, we first determine the fitting parameters $\mu N \Pi_c$ and x_c that allow to collapse all the data one master curve $\chi(x/x_c)$ (Eq. (1) in Methods). Here, (x_c, Π_c) represent the crossover between bubble and sector regime for the reverse cumulative distribution $\Pi(x)$ of clone sizes (Supplementary Fig. 5), and μN is the total number of mutations that entered the population. Because the fitting parameters depend on the population size N, which varied slightly between the colonies (see Supplementary Table 1), we allow the fitting parameters to vary from one colony to another.

The optimal fitting parameters are determined by minimizing the sum of least squares between the reverse cumulative clone size distribution obtained via sequencing and the master curve $\chi(x/x_c)$ derived by rescaling the results from the Eden model simulations with $x_c^{Eden} = 0.068$ and $\Pi_c^{Eden} = 0.019$ (Fig. 4b and Supplementary Fig. 2). Supplementary Table 3 summarizes the fitting parameters for each colony used to generate Fig. 3.

At very low frequencies, which we cannot observe via sequencing because of the limited coverage, we expect a transition from the bubble regime to a different behavior that captures finer scale dynamics (Methods). Although we do not attempt to characterize this regime here, we determine the minimum frequency $x_{\min}$ below which we expect the bubble scaling to fail. We define this frequency as the value at which the extrapolated bubble power-law from the sequencing site frequency spectrum of colonies intercepts the prolonged well-mixed expectation (Supplementary Fig. 2). Its value and the corresponding minimum population size $N_{\min} = N x_{\min}$ is also reported in Supplementary Table 3.

By rescaling frequency x and the number of SNPs $\mu N \Pi(x)$ above frequency x using the parameters in Supplementary Table 3, we can extend the experimental clone size distribution with the Eden model simulations and our theory for different population sizes and mutations rates. We can then compute the mean and the typical number of mutants, and the colony double mutant probability reported in Fig. 5 in the main text. Note that when we sample from the empirical site frequency spectrum to generate Fig. 5, we assume that below $x_{\min}$ the clone size distribution follows a well-mixed distribution.

References

[1] Gnedenko, B. & Kolmogorov, A. Limit distributions for sums of independent random variables. *Amer. J. Math.* **105**, 28–35 (1954).

[2] Krapivsky, P. L., Redner, S. & Ben-Naim, E. *A kinetic view of statistical physics* (Cambridge University Press, 2010).

[3] Kessler, D. A. & Levine, H. Large population solution of the stochastic Luria-Delbruck evolution model. *Proceedings of the National Academy of Sciences of the United States of America* **110**, 11682–7 (2013).

[4] Frank, S. A. & Nowak, M. A. Cell biology: Developmental predisposition to cancer. *Nature* **422**, 494–494 (2003).

[5] Hallatschek, O. & Nelson, D. R. Life at the front of an expanding population. *Evolution* **64**, 193–206 (2010).

[6] Anderson, P. Sensitivity and Resistance to Spectinomycin in Escherichia coli. *Journal of bacteriology* **100**, 939–47 (1969).

[7] Sigmund, C. D., Ettayebi, M. & Morgan, E. A. Antibiotic resistance mutations in 16S and 23S ribosomal RNA genes of Escherichia coli. *Nucleic acids research* **12**, 4653–63 (1984).

[8] Lee, H., Popodi, E., Tang, H. & Foster, P. L. Rate and molecular spectrum of spontaneous mutations in the bacterium escherichia coli as determined by whole-genome sequencing. *Proceedings of the National Academy of Sciences* **109**, E2774–E2783 (2012).

[9] Lynch, M. Estimation of allele frequencies from high-coverage genome-sequencing projects. *Genetics* **182**, 295–301 (2009).
